# Analysis of VOCs in Urine Samples Directed towards of Bladder Cancer Detection

**DOI:** 10.3390/molecules27155023

**Published:** 2022-08-07

**Authors:** Tomasz Ligor, Przemysław Adamczyk, Tomasz Kowalkowski, Ileana Andreea Ratiu, Anna Wenda-Piesik, Bogusław Buszewski

**Affiliations:** 1 Department of Environmental Chemistry and Bioanalytics, Faculty of Chemistry, Nicolaus Copernicus University, 87-100 Toruń, Poland; 2 Interdisciplinary Centre of Modern Technologies, Nicolaus Copernicus University, 87-100 Toruń, Poland; 3 Department of General and Oncologic Urology, Nicolaus Copernicus Hospital in Torun, 87-100 Toruń, Poland; 4 “Raluca Ripan” Institute for Research in Chemistry, Babes-Bolyai University, 30 Fantanele, RO-400239 Cluj-Napoca, Romania; 5 Department of Agronomics, Faculty of Agriculture and Biotechnology, Bydgoszcz University of Science and Technology, 85-796 Bydgoszcz, Poland

**Keywords:** bladder cancer, urine analyses, volatile organic compounds, GC×GC TOF MS

## Abstract

Bladder cancer is one of most common types of cancer diagnosed in the genitourinary tract. Typical tests are costly and characterized by low sensitivity, which contributes to a growing interest in volatile biomarkers. Head space solid phase microextraction (SPME) was applied for the extraction of volatile organic compounds from urine samples, and gas chromatography time of flight mass spectrometry (GC×GC TOF MS) was used for the separation and detection of urinary volatiles. A cohort of 40 adult patients with bladder cancer and 57 healthy persons was recruited. Different VOC profiles were obtained for urine samples taken from each group. Twelvecompounds were found only in the samples from theBC group.The proposed candidate biomarkers are butyrolactone; 2-methoxyphenol; 3-methoxy-5-methylphenol; 1-(2,6,6-trimethylcyclohexa-1,3-dien-1-yl)-2-buten-1-one; nootkatone and 1-(2,6,6-trimethyl-1-cyclohexenyl)-2-buten-1-one.Since most of the studies published in the field are proving the potential of VOCs detected in urine samples for the screening and discrimination of patients with bladder cancer from healthy, but rarely presenting the identity of proposed biomarkers, our study represents a novel approach.

## 1. Introduction

Bladder cancer is one of most common types of neoplasms which invade the genitourinary tract. It is the fourth most common cancer diagnosed in males (after lung, stomach, and prostate cancer) accounting for 380,000 new cases and more than 150,000 deaths worldwide annually. Neoplastic carcinogenesis is associated with molecular and genetic changes in urothelial cells. The main methods of BC treatment are transurethral resection of tumor (in the case of superficial changes caused by the disease) and radical cystectomy in invasive cases. The majority of bladder cancer patients at presentation are staged with a superficial disease (not invading muscularis propria); therefore, they are subject to the transurethral resection of tumor (TURBT) and cystoscopy as the follow up procedure. Cystoscopy is an invasive and expensive procedure, and thus, to perform a sensitive test it is necessary to select a group of patients in which high risk of recurrence balances invasiveness to cost ratio. Tobacco smoke and an occupational exposure to some aromatic amines, mainly 2-naphthylamine, 4-aminobiphenyl, 4,4′-methylenedianiline, and o-toluidine are the main risk factors of bladder cancer development [1]. Various bladder cancer diagnostic tests are used; however, most of them are costly and characterized by low sensitivity. Cystoscopy is a method of choice in the diagnosis of bladder cancer. Currently, numerous studies are being performed to define specific bladder cancer biomarkers. Protein-based urinary markers include a nuclear mitotic apparatus protein 22 (NMP22), bladder tumor antigens (BTA), surviving, and others [2]. However, carbonic anhydrase IX (CAIX) [3,4], which is the 459 amino acid transmembrane protein [5,6], seems to be the most promising diagnostic marker for bladder cancer. Therefore, urine biomarkers are urgentlyneeded when cost-reduction and patient convenience are considered. Many of urinary compounds are metabolic pathways products and might by applied as biomarkers of diseases, especially in metabolic disorders. The well-known examples are trimethylaminuria (fish odor syndrome) and diabetes. Furthermore, bacterial urinary tract infections are frequently recognized due to their uremic odor [7]. Metabolites might be excreted directly into urine by the bladder cancer tissue and can thus change VOC composition in urine. Such an idea originates from experiments in which trained dogs wereused to distinguish people affected by human cancer from those representing the healthy control by sniffing the urine odor [8]. Hence, certain compounds can be identified by chromatography and mass spectrometry. Several research groups analyzed VOCs in urine samples to search for thebiomarkers of different types of cancer (lung, breast, colorectal, leukemia, and lymphoma) [9,10,11]. Other extensive metabolomic studies were focused on genitourinary types of cancer, such as renal cell carcinoma [12,13] and prostate cancer [14].

However, regarding bladder cancer, there have been limited reports on the analyses of VOCs in urine. The earliest study presents only the determination of formaldehyde in the headspace of urine [15]. Jobu et al. used a needle trap device (NTD) and gas chromatography mass spectrometry (GC–MS) for the identification of VOCs in urine of patients affected by bladder cancer and found potential markers [16]. More recently, Pinto et al. applied GC–MS for searching of potential biomarkers of BC [17]. They used different SPME fibers for the extraction of VOCs and volatile carbonyl compounds (VCCs), respectively. The proposed set of markers showed 70% sensitivity and 89% specificity for BC detection.A study on the recognition of bladder cancer from urine headspace was conducted by Cauchi et al. [18]. They found elevated concentrations of hexanal, benzaldehyde, butyrophenone, 3-hydroxyanthranilic acid, benzoic acid, trans-3-hexanoic acid, cis-3-hexanoic acid, and 2-butanone in the samples from patients with BC. Their methodology could allow for the discrimination of 72 patients with BC from 46 healthy controls with 90% sensitivity and 88% specificity. Moreover, sensor technology for the detection of VOCs in urine for BC recognition can be also mentioned. Sensor devices are relatively cheap, simple, and enable rapid measurement. However, sensors are useful for recognizing VOC patterns in a sample headspace but not for identifying unknowns. Zhu et al. used optical sensory arrays to detect the urinary bladder cancer-related VOCswith 77.75% sensitivity and 93.25% specificity [19]. Heers et al. applied commercially available Cyranose for the measurement of VOCs in urine. In the above mentioned study, 93.3% sensitivity and 86.7% specificity were reported for the discrimination of BC from healthy controls [20]. In recent years, attention has been focused on metabolomics for the detection of BC markers. Rodrigues et al. presented a study in which metabolic alterations between low-grade and high-grade BC cultured cells were discussed. They observed different levels of myristic, palmitic, and palmitoleic acids in high-grade BC in comparison to low-grade BC grade [21]. In another study, they identified VOCs released from bladder cancer cell lines. Generally, the levels of ketones and alkanes have been increased in BC in comparison to those found in normal cells [22]. Lin et al. identified 922 compounds in urine from BC patients and further proposed eightputative biomarkers of BC, mainly carbohydrates [23]. Pasikanti et al. performed urinary metabotyping and proposed 46 metabolites that characterize BC. The method demonstrates 71% sensitivity and 100% specificity in the detection of BC [24]. Zhou et al. developed a pseudo-targeted method and found 76 specific metabolites in urine, which differentiate BC from healthy controls [25]. Such complicated metabolomic investigations require different analytical platforms, especially chromatographic techniques and mass spectrometry. In order to find potential BC biomarkers, multiplatform urinary metabolomics have been applied. In these studies, urine was collected from 24 patients with BC and 24 healthy people. Samples were analyzed by HPLC-TOF/MS, GC MS/MS and 1H NMR. Seventeenmetabolites in urine were classified as compounds, the presence of which allows the effectively differentiation between people affected by BC and controls [26]. Recently, Gould et al. reviewed analytical techniques for the detection of volatile compounds from the human body with an emphasis put on GC×GC analysis [27].

Therefore, developing a non-invasive method for screening of bladder cancer is desirable. The purpose of this research is to identify VOCs in urine samples and to find compounds, which discriminate patients with bladder cancer and healthy controls. In this study, the HS-SPME GC×GC TOF-MS method was developed for the analysis of VOCs in urine. A cohort of 40 adult patients with bladder cancer and 57 healthy persons was recruited. The compounds were separated using a polar (Rtx-WAX) and a non-polar (Rtx-5 MS) column combination. The experiments were performed by means of low resolution TOF MS.

As the results of previous studies in the field proved, VOCs detected in urine samples have potential for screening, discriminating, and detecting bladder cancer. There are studies presenting altered levels of VOCs in patients with bladder cancer and others reporting discrimination between various diseased patient groups and controls using statistical tools; however, certain proposed markers elevated in bladder cancer or appearing in bladder cancer patient groupswerenot often presented. In our study, we discovered twelveVOCs that occurred in patients with bladder cancer only and six that highlighted elevated levels in bladder cancer group compared with controls, and therefore the present study represents an original and valuable approach in the field.

## 2. Results

The peak areas of compounds were used to prepare a matrix for chemometric analyses, and a dataset representing the distributions of 373 compounds in all the urine samples was constructed. Compounds detected in urine showed a huge chemical diversity: ketones, alcohols, lactones, heterocyclic compounds, volatile sulfur compounds, etc. Compounds typically used in cosmetics, mainly hexylcinnamal, amylcinnamal, galaxolide, and Iso E Super, constituted a separate group of identified substances. We excluded these compounds due to their exogenous origin. The evaluation of chromatographic data was conducted following an especially designed three-step procedure established by the design of three step procedure. In the first step, data reductions were carried out according to the principle of elimination, i.e., by the elimination of those variables which appeared in less than fivesamples in individual groups (patients/healthy). This reduced the number of variables from 373 to 122. Afterwards, a meta-analysis was applied to prepare the matrix of compounds which could be selected as primary biomarkers (n = 12). These ones were found only in the group of patients with cancer (Table 1). The data matrix was initially normalized prior to analyses due to huge differences in peak areas of the investigated compounds.

The remaining compounds (n = 110) were evaluated by the Kolmogorov–Smirnov test to check significant differences (*p* <0.05) of each peak area between the healthy group and the BC patients (n = 33) (Table 2). The Kolmogorov–Smirnov test was applied to verify the hypothesis that each chemical compound was taken from a different part of population (BC vs. healthy). Large differences in the dispersion and skewness of the distributions of these compounds justify the choice of the K–S test. In Table 2, VOCs are listed in the order from the highest to the lowest level of significance.

**Figure 1:**
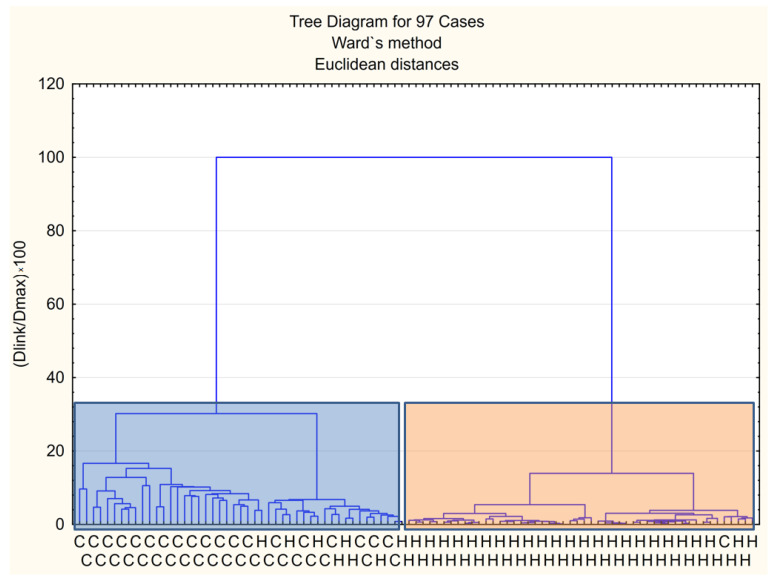
Dendrogram based on the cluster analysis.

**Figure 2:**
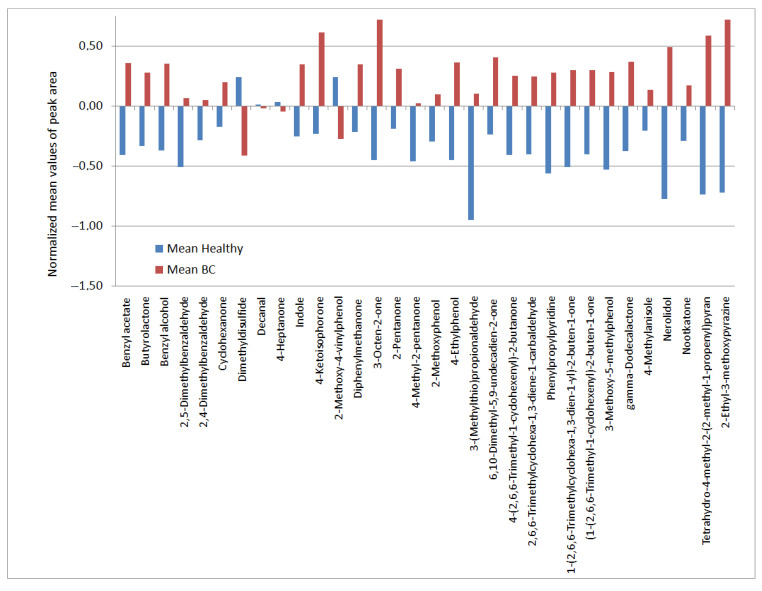
Normalized mean peak areas of compounds for healthy persons (H) and cancer patients(BC).

Figure 1 shows a dendrogram based on a cluster analysis (containing 32 compounds listed in Table 2), which was developed in order to obtain a preliminary exploration of the data. Two clusters are observed. Patients were classified on the basis of grouping the volatile compounds, except sevenhealthy volunteers who were wrongly classified to the left cluster, and only one patient with cancer was misclassified. This simple analysis caused us to apply more sophisticated methods of classification. Moreover, within the further meta-analysis, we observed that in healthy people some compounds are present at levels higher than in patients with cancer (Figure 2). This effect was observed for four compounds, namely, 4-heptanone, dimethyldisulfide, decanal, and 2-methoxy-4-vinylphenol. They can be assigned as “negative markers”.

**Figure 3:**
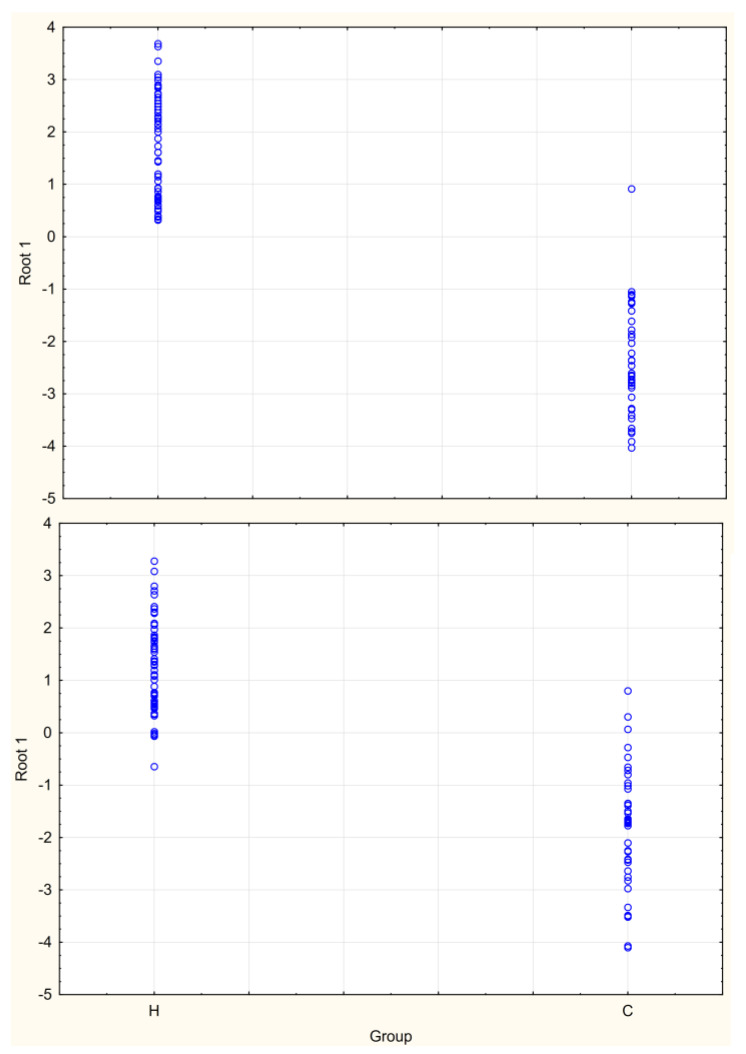
Canonical root scores in both groups, where the upper graph consists of “negative markers” and the lower consists of only positive markers. H—healthy persons and BC—cancer patients.

The discriminant analysis followed by the computation of canonical roots was performed with and without “negative markers”. The backward stepwise analysis was chosen. Eighthealthy people and sevencancer patients were exuded from the building of the classifier. Those cases were used to cross-validate the model. In the first option, discriminant functions were computed more efficiently than in the second one (Wilk′s Lambda equal to 0.197 and 0.294). The canonical analysis revealed that only one root is necessary to discriminate between both groups (Figure 3). One cancer patient was misclassified (the case of the model building) when all the compounds were used for the data analysis, in contrast to the second version, in which some cases of the both groups overlapped each other within a certain range (threeand four cases from the heathy and BC cross-validation groups, respectively).

Artificial neural networks (ANN) turned out to be the best method of classification. The multilayer perceptron topology was chosen. The data matrix was divided into three sets: training, validation, and testing (50%, 25%, and 25% cases, respectively), and 200 different topologies were tested. The BFGS training algorithm was used. The best ANN consisted of 27 neurons in the input layer (equal to the number of compounds), 17 neurons in the hidden layer with the logistic activation function and 2 as the outputs with the identity activation function. All the cases were classified correctly. We selected candidate biomarkers, which found at least 50% of BC patients for whom the fold change is greater than 5 and with *p* < 0.001, namely butyrolactone, 2-methoxyphenol, 3-methoxy-5-methylphenol, 1-(2,6,6-trimethylcyclohexa-1,3-dien-1-yl)-2-buten-1-one, nootkatone, and 1-(2,6,6-trimethyl-1-cyclohexenyl)-2-buten-1-one. These substances are promising candidates for BC markers in urine.

## 3. Discussion

We developed a methodology for the identification of VOCs in urine samples to distinguish patients with bladder cancer from the healthy group. Comprehensive gas chromatography and mass spectrometry (GC×GC TOF MS) is a very effective technique in the search forbiomarkers and enables the analysis and processing of huge amounts of samples. Generally, more than 100 chromatographic signals from different substances were present in each sample. Such a large variety of compounds significantly hindered the classification of the substances and the selection of potential biomarkers. Ketones and terpenes were mostly observed in urine. Plant derived food is the main source of terpenes in urine. However, the endogenous origin of terpenes cannot be excluded [28,29]. In all the samples, a total of 373 substances were identified by means of HS SPME GC×GC TOF MS. However, optimized liquid–liquid extraction for GC–MS enabled the detection of more than 330 VOCs in urine samples [30]. Usually, it was revealed that urine samples taken from patients contained a larger number of components. Aliphatic aldehydes and alkanes, mainly ethane and pentane, are produced in lipid oxidation processes. In general, their concentration increases during inflammation and oxidative stress [31] since they excessively produce reactive oxygen species (ROS). In the case of malignant tumors, patients may experience inflammation caused by cancer. We observed a slight decrease in the decanal level in urine samples taken from patients diagnosed with BC in comparison with the samples from the control group. Similar tendencies have also been noticed in the case of patients affected by prostate cancer [14]. The presence of aldehydes resulting from alcohols and fatty acids metabolism are also formed during the catabolic processes of amino acids and carbohydrates. They are known as the biomarkers of oxidative stress. From among the substances which were present at higher concentrations in patients, 2-pentanone (*p* < 0.025) should be mentioned. This compound was also found in urine samples taken from patientsaffected by renal cell carcinoma or prostate cancer [13,14]. Increased levels of both ketones as well as alcohols were observed in diabetes [13]. Hence, diabetes should be considered as one of the confounding factors. Assuming that ketones result from fatty metabolism, their raised level may be caused by the increased oxidation rate of fatty acids and glycol-oxidation. Regarding a significantly higher level of 2,5-dimethylbenzaldehyde (*p* < 0.025) observed in our research, the fact that the substance was also identified in the case of prostate cancer should be mentioned. Therefore, this aldehyde is thus not specific to BC. The oxidation of alcohols or fatty acids metabolism can also be its source [14]. As our work showed, 4,6-dimethyl-2-heptanone occurred exclusively in patients suffering from BC, i.e., it was not present in healthy individuals. Thisis in contradiction with other researchers′ findings as they observed this ketone both in patients as well as healthy people. They, however, noticed a decrease of 4,6-dimethyl-2-heptanone in patients when compared to those in the control. This ketone was also detected in patients affected by prostate cancer [14]. Considering other substances detected, including 2-pentanone, 4-heptanone, dimethyl disulphide, and butyrophenone, they were denoted as possible markers of BC by Cauchi and coworkers [18]. Lower levels of dimethyl disulfide, decanal, 4-heptanone, and 2-methoxy-4-vinylphenol in urine persons suffering from BC may be attributed to the downregulations of protein metabolism and the ketogenic pathway caused by cancer. Extremely important research aimed at identifying and characterizing VOCs excreted by cultured BC cells was performed by Rodrigues and coworkers [22]. They identified important cell metabolites, such as 2-pentadecanone, dodecanal, and *γ*-dodecalactone. They also confirmed the presence of cyclohexanone. From the above-mentioned substances, as a result of our studies, we were able to confirm the presence of *γ*-dodecalactone and cyclohexanone in urine of patients with BC, which undeniably proves that cancerous cells are their source. Increased amounts of cyclahexanone have also been detected in the case of prostate cancer.

We selected candidate biomarkers, which were found in at least 50% of BC patients and for whom the fold change is greater than 5 and with *p* < 0.001, namely, butyrolactone, 2-methoxyphenol,3-methoxy-5-methylphenol, 1-(2,6,6-trimethylcyclohexa-1,3-dien-1-yl)-2-buten-1-one, nootkatone, and 1-(2,6,6-trimethyl-1-cyclohexenyl)-2-buten-1-one. Oxidative processes are an important factor in cancer diseases. On the other hand, hypoxia is a very common situation which results from the rapid growth of bladder cancer. Carbonic anhydrases (CA) allow cancerous cells to survive under hypoxic conditions. Carbonic anhydrase IX (CA IX) regulates cell proliferation and adhesion as well as tumor progression. The expression of CA IX is observed in BC. Moreover, CA IX can inhibit the oxidation of several compounds. We expected that elevated concentrations of2-methoxyphenol, 3-methoxy-5-methylphenol,1-(2,6,6-trimethylcyclohexa-1,3-dien-1-yl)-2-buten-1-one, nootkatone, and 1-(2,6,6-trimethyl-1-cyclohexenyl)-2-buten-1-onein urine are due to the inhibition of oxidation of these substances by CA IX and/or hypoxic conditions inside the cancerous cells. Moreover, exfoliated cancer cells which are observed in the urine of patients with BC release VOCs, which could facilitate detection of the markers. This hypothesis will constitute the subject of another study. However, finding the VOCs biosynthesis paths in an organism is a very important but also an extremely difficult research task. Such studies require carrying out model reactions and using isotope-labelled substrates.

The relatively small group of participants is the major limitation of the study. A larger group is necessary to validate our results and biomarker candidates. Moreover, high variability in the presence of individual substances and chemical classes of compounds in urine samples is observed. Therefore, a more general model should consider the proposed volatiles candidates and biochemical dependences among them. In addition, diet, medication metabolites, and exogenous compoundsare important confounding factors for urinary biomarkers. Moreover, knowledge regarding VOC metabolism in human cells is very limited.

## 4. Materials and Methods

### 4.1. Materials

An SPME automatic holder with CAR/PDMS fiber was purchased from Supelco (Bellefonte, PA, USA). Screw-top headspace glass vials with silicon/PTFE septa and caps were supplied by Supelco. Ultra-high purity helium BIP 5.5 was purchased from Air Liquide (Bydgoszcz, Poland). Sodium chloride was purchased from Sigma-Aldrich (Steinheim, Germany).

### 4.2. Apparatus

The analysis was carried out using an Agilent 6890 gas chromatograph (Agilent Technologies, Waldbronn, Germany) connected to a Leco Pegasus 4D GC×GC TOF-MS mass spectrometer (Leco Corp., St. Joseph, MO, USA). The gas chromatograph was equipped with a split/splitless injector with a 0.75 mm ID liner, two ovens, a dual stage jet cryogenic modulator, and an MPS-2 autosampler, (Gerstel, Mulhheim, Germany) for automatic SPME extraction and desorption. The first dimension was Rtx-Wax column with an integrated 5 m precolumn (35 m × 0.25 mm × 0.20 μm), and the second dimension was an Rtx-5 MS column (1.5 m × 0.18 mm × 0.20 µm), both supplied by Restek (Bellefonte, PA, USA). The injector was set to 230 °C. SPME desorptions were performed in the splitless mode within 1 min. Helium was used as the carrier gas in the constant flow mode at 1 mL/min. The mass spectrometer operatedin EI (70 eV) mode. The ion source temperature and transfer line were set to 225 °C; acquisition frequency 180 Hz; mass range 35–350 amu. The first dimension column temperature was programmed as follows: the initial temperature of 40 °C was held for 3 min, then the temperature was increased by 10 °C/min to 235 °C and maintained for 5 min at this value. The second dimension column temperature was maintained 5 °C higher than the corresponding first dimension column. The modulator temperature was maintained 15 °C higher than the corresponding one of the second dimension column. The temperature rates and hold times were the same for the two columns and the modulator. Chromatof (Leco Corp., St. Joseph, MO, USA) software version 4.50.8.0 was used for data acquisition and processing. The identification of volatile metabolites was performed on the basis of similarity of the measured mass spectra to MS libraries if the match factor was higher than 850 and signal-to-noise (S/N) ratio was higher than 25. The unique mass for each peak was chosen by the software algorithm and applied for deconvolution and peak area calculations. For the purpose of identification, the mass spectrum of each compound was automatically matched to those in the MS library, Wiley 9-th Ed./NIST 2011. For urine normalization, we selected patients and healthy individualsfor experiments if the specific gravity of urine ranged from 1010 to 1030.

### 4.3. Data Processing and Identification

Data processing parameters were as follows: tune check on, baseline offset 1, peak width 2 s for 2nd dimension, match spectra required to combine 850, minimum S/N 25 subpeak to retain andthe maximum number of unknown peaks to find 10,000. The unique mass for each peak was chosen by an Chromatof algorithm and applied for deconvolution and peak area calculations. An automatic peak finding and deconvolution S/N = 5, library search mode-forward, library identify-normal, maximum mass 350, library mass threshold 5, and minimum similarity match 850. The QC method was used for the mass spectrometer, and the instrument was adjusted to optimize mass 219 *m*/*z*, with the minimum intensity of 20,000 and the maximum intensity of 100,000. The Chromatof (Leco) software version 4.50.8.0 was used for data acquisition and processing. For the purpose of identification, the mass spectrum of each compound was automatically matched to those in the MS library, Wiley 9-th Ed./NIST 2011. The automatically identified substances were manually verified in order to provide the minimum score of 900, remove artifacts such as silicones, column bleeding, and comparison with literature data describing the substances detected in the urine headspace.

### 4.4. Sample Preparation

Urine samples were immediately frozen and stored at −20 °C. Prior to analyses, the samples were defrosted and then prepared as follows: NaCl (3.6 g) was weighed in a 20 mL glass vial, and 10.0 mL of urine sample was added. Then, the vial was capped with a PTFE/Silicone septum and a screw cap. Each vial was incubated for 30 min at 45 °C. The fiber was exposed to the headspace at 45 °C for 45 min. After the sampling, SPME fiber was desorbed in a hot GC inlet for 1 min in the splitless mode. SPME extraction, desorption, and thermal fiber cleaning were carried out automatically by means of an MPS-2 autosampler. The fiber and sample conditions were applied according to those utilized in earlier experiments [32].

### 4.5. Human Subjects

Study design, patients, and data collection:

This prospective study involved data from 40 consecutive patients undergoing the transurethral resection of bladder tumor. In all the cases, bladder cancer was confirmed by a histopathologic examination. The patients were recruited at the Department of General and Oncologic Urology, Nicolaus Copernicus Hospital in Torun, Poland.

Early morning mid-stream urine samples were collected in 100 mL sterile plastic containers at the hospital. In the case of healthy volunteers, urine samples were self-collected at home in the same manner. Samples were immediately frozen at the hospital or laboratory and stored at −20°C. Prior to analyses, the samples were defrosted. All the patients underwent a preoperative examination, including routine laboratory tests, a chest radiogram, and an abdominal ultrasonography scan. In each case, a transurethral resection was performed, and a histopathologic report which confirmed cancer was obtained. Oncological variables and results were noted, and neoplasm staging was carried out according to the TNM classification system. Age, gender, co-morbidities, surgical history, and laboratory test results were collected. The demographic data are presented in Table 3. We selected a cohort of 40 adult patients with BC. The average age was 59.9 years old. The enrolled healthy group consisted of 57 people. The patients and the controls’ diet was not particularly restricted.

### 4.6. Ethical Considerations

All subjects had given their informed consent for inclusion before they participated in the study. The procedures were performed in accordance with the ethical standards of the ethics committee of the Nicolaus Copernicus University (number approval No. KB 201/2016 from 22 March 2016) and with the Declaration of Helsinki (1964) and its later amendments or comparable ethical standards.

## 5. Conclusions

Comprehensive GC×GC TOF MS allows the separating and finding of hundreds of signals in each urine sample. A great variety was observed in the presence of individual chemical substances and classes of compounds. However, the origin of the majority of VOCs detected in urine still remains unknown.

We selected candidate biomarkers, which found at least 50% of BC patients and for whom the fold change is greater than 5 and with *p* < 0.001, namely butyrolactone, 2-methoxyphenol,3-methoxy-5-methylphenol, 1-(2,6,6-trimethylcyclohexa-1,3-dien-1-yl)-2-buten-1-one, nootkatone, and 1-(2,6,6-trimethyl-1-cyclohexenyl)-2-buten-1-one.Such compounds are promising candidates for VOC model BC markers in urine.

## Figures and Tables

**Table 1:** Selection of compounds occurring only in the group of patients with bladder cancer.

Compound	Origin *	Presence in BC Group	% of Occurrence in BC Group
Pyridine	exogenous, food	22	55
4-Propylbenzaldehyde	-	19	47.5
2,3-Heptanedione	exogenous, food	18	45
Heptanal	endogenous/exogenous, food	18	45
4-Methyl-3-pentenoic acid	-	14	35
1-Heptanol	exogenous, food	13	32.5
1-Octen-3-one	exogenous, food, esp. mushrooms	12	30
2,6-Dimethyaniline	exogenous, lidocaine metabolism	11	27.5
11-Hexadecen-1-ol	-	10	25
4,6-Dimethyl-2-heptanone	-	9	22.5
2-Butyl-1-octanol	-	8	20
2,3,5-Trimethylfuran	exogenous, food	8	20

* The Human Metabolome Database information.

**Table 2:** Results of the Kolmogorov–Smirnov test.

Compound	*p*-Value	Mean Peak Area for Healthy Group	Mean Peak Area for BC Group	Fold Change	Valid Healthy (n = 57)	Valid BC (n = 40)
Butyrolactone	*p* < 0.001	136,261	974,481	7.2 ↑	22	26
4-Heptanone	*p* < 0.001	554,774	431,521	0.8 ↓	39	28
2-Methoxyphenol	*p* < 0.001	29,899	826,976	27.7 ↑	9	27
6,10-Dimethyl-5,9-undecadien-2-one	*p* < 0.001	35,908	793,666	22.1 ↑	31	18
1-(2,6,6-Trimethylcyclohexa-1,3-dien-1-yl)-2-buten-1-one	*p* < 0.001	12,923	134,868	10.4 ↑	13	22
1-(2,6,6-Trimethyl-1-cyclohexenyl)-2-buten-1-one	*p* < 0.001	8235	48,021	5.8 ↑	15	20
3-Methoxy-5-methylphenol	*p* < 0.001	11,505	106,669	9.3 ↑	14	26
Nerolidol	*p* < 0.001	9752	45,975	4.7 ↑	14	22
Nootkatone	*p* < 0.001	10,112	78,722	7.8 ↑	13	22
Tetrahydro-4-methyl-2-(2-methyl-1-propenyl)pyran	*p* < 0.001	13,604	53,870	4.0 ↑	12	15
Dimethyldisulfide	*p* < 0.005	222,020	36,063	0.2 ↓	41	24
Decanal	*p* < 0.005	208,545	193,279	0.9 ↓	42	32
3-Octen-2-one	*p*< 0.005	28,212	234,471	8.3 ↑	16	10
4-Ethylphenol	*p* < 0.005	15,607	76,793	4.9 ↑	13	16
4-Methylanisole	*p* < 0.005	7566	3,223,318	426.0 ↑	10	15
2-Ethyl-3-methoxypyrazine	*p* < 0.005	4986	42,007	8.4 ↑	9	9
2,6,6-Trimethylcyclohexa-1,3-diene-1-carbaldehyde	*p* < 0.01	20,175	36,846	1.8 ↑	13	21
gamma-Dodecalactone	*p* < 0.01	14,092	71,939	5.1 ↑	12	12
Benzyl acetate	*p* < 0.025	30,275	185,006	6.1 ↑	8	9
Benzyl alcohol	*p* < 0.025	35,203	135,474	3.8 ↑	22	23
2,5-Dimethylbenzaldehyde	*p* < 0.025	3539	107,940	30.5 ↑	3	22
2,4-Dimethylbenzaldehyde	*p* < 0.025	7563	1,902,422	251.5 ↑	3	16
4-Ketoisophorone	*p* < 0.025	38,371	115,927	3.0 ↑	16	6
2-Methoxy-4-vinylphenol	*p* < 0.025	379,425	64,421	0.2 ↓	28	25
2-Pentanone	*p* < 0.025	12,236,224	28,715,105	2.3 ↑	41	25
Cyclohexanone	*p* < 0.05	121,526	2,480,962	20.4 ↑	22	19
Indole	*p*< 0.05	45,452	127,509	2.8 ↑	40	29
Diphenylmethanone	*p* < 0.05	31,987	77,544	2.4 ↑	21	13
4-(2,6,6-trimethyl-1-cyclohexenyl)-2-butanone	*p* < 0.05	21,009	83,966	4.0 ↑	15	24
Phenylpropylpyridine	*p* < 0.05	15,226	37,591	2.5 ↑	10	20
4-Methyl-2-pentanone	-	24,972	595,665	23.9 ↑	1	17
3-(Methylthio)propionaldedyde	-	14,300	32,921	2.3 ↑	1	9

**Table 3:** Demographic data.

	Patients	Controls
Number of persons	40	57
Female	10	18
Male	30	39
Number of smokers	24	27
Ex-smokers	6	5
Non-smokers	10	25
Average age	59.9	55.8
Median age	58.5	50.2
SD	10.6	12.74
Comorbidities	hypertension (4) heart failure (2) arrhythmia (1)	hypertension (5)
Medications	bis (3), carv (1) ind/per (2), aml (1) met + ami (1)	bis (2), car (1), per (1) aml (1)

Abbreviations: bis—bisoprolol; carv—carvedilol; ind/per—indapamide + perindopril; aml—amlodipine; met—metoprolol; ami—amiodarone; number of patients in the parenthesis.

## Data Availability

Data arecontained within the article.

## References

[1] Cumberbatch M.G.K., Jubber I., Black P.C., Esperto F., Figueroa J.D., Kamat A.M., Kiemeney L., Lotan Y., Pang K., Silverman D.T. (2018). Epidemiology of Bladder Cancer: A Systematic Review and Contemporary Update of Risk Factors in 2018. Eur. Urol..

[2] Xylinas E., Kluth L.A., Rieken M., Karakiewicz P.I., Lotan Y., Shariat S.F. (2014). Urine markers for detection and surveillance of bladder cancer. Urol. Oncol. Semin. Orig..

[3] Malentacchi F., Vinci S., Melina A.D., Kuncova J., Villari D., Giannarini G., Nesi G., Selli C., Orlando C. (2012). Splicing variants of carbonic anhydrase IX in bladder cancer and urine sediments. Urol. Oncol. Semin. Orig..

[4] De Martino M., Lucca I., Mbeutcha A., Wiener H.G., Haitel A., Susani M., Shariat S.F., Klatte T. (2015). Carbonic anhydrase ix as a diagnostic urinary marker for urothelial bladder cancer. Eur. Urol..

[5] Wichert M., Krall N. (2015). Targeting carbonic anhydrase IX with small organic ligands. Curr. Opin. Chem. Biol..

[6] Klatte T., Seligson D.B., Rao J.Y., Yu H., de Martino M., Kawaoka K., Wong S.G., Belldegrun S., Pantuck A.J. (2009). Carbonic anhydrase IX in bladder cancer. Cancer.

[7] Buljubasic F., Buchbauer G. (2015). The scent of human diseases: A review on specific volatile organic compounds as diagnostic biomarkers. FlavourFragr. J..

[8] Cornu J.N., Cancel-Tassin G., Ondet V., Girardet C., Cussenot O. (2011). Olfactory detection of prostate cancer by dogs sniffing urine: A step forward in early diagnosis. Eur. Urol..

[9] Antón A.P., Ramos Á.G., del Nogal Sánchez M., Pavón J.L.P., Cordero B.M., Pozas Á.P.C. (2016). Headspace-programmed temperature vaporization-mass spectrometry for the rapid determination of possible volatile biomarkers of lung cancer in urine. Anal. Bioanal. Chem..

[10] Silva C.L., Passos M., Camara J.S. (2011). Investigation of urinary volatile organic metabolites as potential cancer biomarkers by solid-phase microextraction in combination with gas chromatography-mass spectrometry. Brit. J. Cancer.

[11] Silva C.L., Passos M., Câmara J.S. (2012). Solid phase microextraction, mass spectrometry and metabolomic approaches for detection of potential urinary cancer biomarkers—A powerful strategy for breast cancer diagnosis. Talanta.

[12] Monteiro M., Carvalho M., Henrique R., Jerónimo C., Moreira N., de Lourdes Bastos M., Guedes de Pinho P. (2014). Analysis of volatile human urinary metabolome by solid-phase microextraction in combination with gas chromatography–mass spectrometry for biomarker discovery: Application in a pilot study to discriminate patients with renal cell carcinoma. Eur. J. Cancer.

[13] Monteiro M., Moreira N., Pinto J., Pires-LuısRHenrique A.S., Jerónimo C., de Lourdes Bastos M., Gil A.M., Carvalho M., GuedesndePinho P. (2017). GC-MS metabolomics-based approach for the identification of a potential VOC-biomarker panel in the urine of renal cell carcinoma patients. J. Cell. Mol. Med..

[14] Lima A.R., Pinto J., Azevedo A.I., Barros-Silva D., Jerónimo C., Henrique R., de Lourdes Bastos M., Guedes de Pinho P., Carvalho M. (2019). Identification of a biomarker panel for improvement of prostate cancer diagnosis by volatile metabolic profiling of urine. Br. J. Cancer.

[15] Spanel P., Smith D., Holland T.A., Al Singary W., Elder J.B. (1999). Analysis of formaldehyde in the headspace of urine from bladder and prostate cancer patients using selected ion flow tube mass spectrometry. Rapid Commun. Mass Spectrom..

[16] Jobu K., Sun C., Yoshioka S., Yokota J., Onogawa M., Kawada C., Inoue K., Shuin T., Sendo T., Miyamura M. (2012). Metabolomics study on the biochemical profiles of odor elements in urine of human with bladder cancer. Biol. Pharm. Bull..

[17] Pinto J., Carapito Â., Amaro F., Lima A.R., Carvalho-Maia C., Martins M.C., Jerónimo C., Henrique R., de Lourdes Bastos M., Guedes de Pinho P. (2021). Discovery of volatile biomarkers for bladder cancer detection and staging through urine metabolomics. Metabolites.

[18] Cauchi M., Weber C.M., Bolt B.J., Spratt P.B., Bessant C., Turner D.C., Willis C.M., Britton L.E., Turner C., Morgan G. (2016). Evaluation of gas chromatography mass spectrometry and pattern recognition for the identification of bladder cancer from urine headspace. Anal. Methods.

[19] Zhu S., Corsetti S., Wang Q., Li C., Huang Z., Nabi G. (2019). Optical sensory arrays for the detection of urinary bladder cancer-related volatile organic compounds. J. Biophotonics.

[20] Heers H., Gut J.M., Hegele A., Hofmann R., Boeselt T., Hattesohl A., Koczulla A.R. (2018). Non-invasive detection of bladder tumors through volatile organic compounds: A pilot study with an electronic nose. Anticancer Res..

[21] Rodrigues D., Pinto J., Araújo A.M., Jerónimo C., Henrique R., de Lourdes Bastos M., Guedes de Pinho P., Carvalho M. (2019). GC-MS metabolomics reveals distinct profiles of low- and high-grade bladder cancer cultured cells. Metabolites.

[22] Rodrigues D., Pinto J., Araújo A.M., Monteiro Reis S., Jerónimo C., Henrique R., de Lourdes Bastos M., Guedes de Pinho P., Carvalho M. (2018). Volatile metabolomic signature of bladder cancer cell lines based on gas chromatography–mass spectrometry. Metabolomics.

[23] Lin J.Y., Juo B.R., Yeh Y.H., Fu S.H., Chen Y.T., Chen C.L., Wu K.P. (2021). Putative markers for the detection of early-stage bladder cancer selected by urine metabolomics. BMC Bioinform..

[24] Pasikanti K.K., Esuvaranathan K., Hong Y., Ho P.C., Mahendran R., Mani L.R.N., Chiong E., Chan E.C.Y. (2013). Urinary metabotyping of bladder cancer using two-dimensional gas chromatography time-of-flight mass spectrometry. J. Proteome Res..

[25] Zhou Y., Song R., Ma C., Zhou L., Liu X., Yin P., Zhang Z., Sun Y., Xu C., Lu X. (2017). Discovery and validation of potential urinary biomarkers for bladder cancer diagnosis using a pseudotargeted GC-MS metabolomics method. Oncotarget.

[26] Jacyna J., Wawrzyniak R., Balayssac S., Gilard V., Malet-Martino M., Sawicka A., Kordalewska M., Nowicki Ł., Kurek E., Bulska E. (2019). Urinary metabolomic signature of muscle-invasive bladder cancer: A multiplatform approach. Talanta.

[27] Gould O., Drabińska N., Ratcliffe N., de Lacy Costello B. (2021). Hyphenated mass spectrometry versus real-time mass spectrometry techniques for the detection of volatile compounds from the human body. Molecules.

[28] Amann A., de Lacy Costello B., Miekisch W., Schubert J., Buszewski B., Pleil J., Ratcliffe N., Risby T. (2014). The human volatilome: Volatile organic compounds (VOCs) in exhaled breath, skin emanations, urine, feces and saliva. J. Breath Res..

[29] Drabińska N., Flynn C., Ratcliffe N., Belluomo I., Myridakis A., Gould O., Fois M., Smart A., Devine T., Costello B.D.L. (2021). A literature survey of all volatiles from healthy human breath and bodily fluids: The human volatilome. J. Breath Res..

[30] Drabińska N., Młynarz P., de Lacy Costello B., Jones P., Mielko K., Mielnik J., Persad R., Ratcliffe N.M. (2020). An optimization of liquid–liquid extraction of urinary volatile and semi-volatile compounds and its application for gas chromatography–mass spectrometry and proton nuclear magnetic resonance spectroscopy. Molecules.

[31] Ratcliffe N., Wieczorek T., Drabińska N., Gould O., Osborne A., De Lacy Costello B. (2020). A mechanistic study and review of volatile products from peroxidation of unsaturated fatty acids: An aid to understanding the origins of volatile organic compounds from the human body. J. Breath Res..

[32] Ligor T., Zawadzka J., Strączyński G., González Paredes R.M., Wenda-Piesik A., Ratiu I.A., Muszytowski M. (2021). Searching for potential markers of glomerulopathy in urine by HS-SPME-GC×GC TOFMS. Molecules.

